# Application of a Combined Transmittance/Fluorescence Leaf Clip Sensor for the Nondestructive Determination of Nitrogen Status in White Cabbage Plants

**DOI:** 10.3390/s21020482

**Published:** 2021-01-12

**Authors:** Stanisław Kaniszewski, Artur Kowalski, Jacek Dysko, Giovanni Agati

**Affiliations:** 1 Research Institute of Horticulture, Konstytucji 3Maja 1/3, 96-100 Skierniewice, Poland; stanislaw.kaniszewski@inhort.pl (S.K.); artur.kowalski@inhort.pl (A.K.); jacek.dysko@inhort.pl (J.D.); 2 Istituto di Fisica Applicata “Nello Carrara”-CNR, Via Madonna del Piano, 10-50019 Sesto Fiorentino (FI), Italy

**Keywords:** *Brassica oleracea* L. var. *capitata* subvar. *alba*, chlorophyll fluorescence, flavonols, nitrogen, optical sensor, precision fertilization

## Abstract

The correct fertilization of vegetable crops is commonly determined on the basis of soil and plant costly destructive analyses, demanding more sustainable non-invasive optical detection. Here, we tested the ability of the combined transmittance/fluorescence leaf clip Dualex device for determining the nitrogen (N) status of cabbage plants. Fully developed leaves from plants grown under different N rates of 0; 100; 200; 300 kg N ha^−1^ in 2018 and 2019 were measured in the field by the Dualex sensor twice a year in July and October. The chlorophyll (Chl) and nitrogen (nitrogen balance index, NBI) indices and the flavonols (Flav) index of the sensor were positively and negatively correlated to leaf nitrogen, respectively. Merging the two-years data, the NBI versus leaf N correlation was less point dispersed in October than July (R^2^ = 0.76 and 0.64, respectively). NBI was also correlated to cabbage yield, better in July than October. Our results showed that the multiparametric Dualex device can be used as precision agriculture tool for the early prediction of plant N and cabbage yield with economic advantage for the growers and reduced environmental contamination due to nitrate leaching.

## 1. Introduction

Soil analysis is one way to determine pre-plant fertilizer doses and fertilizer recommendations [1]. Plant analysis, or more precisely the analysis of the indicator part of the plant, is used to confirm the correctness of the pre-plant doses used [2]. Plant analysis carried out at the appropriate plant growth stage allows fertilization correction, especially for more labile components such as nitrogen (N). Soil and plant analysis can assess the adequacy of N management, identify deficient, adequate and excessive crop N status. This is particularly important for crops with a high nitrogen demand as white head cabbage (*Brassica oleracea* var. *capitata* subvar. *alba* L.).

For that, considering the mineral N present in the 0–60 cm soil layer at planting, fertilization rates between about 130 and 270 kg N ha^−^^1^, were recommended [3]. A precise determination of the plant N status can be also fundamental to achieve high quality levels of cabbage heads, with limited content of nitrates, and reduce the risk of ground water nitrogen pollution [4].

Plant N is usually measured by time-consuming destructive analyses [5] of a limited number of plant samplings that may not be fully representative of an entire field crop. Lately, however, there has been an increasing trend to adopt non-destructive methods by using optical sensors to assess the nutritional nitrogen status of plants [6,7]. They provide a rapid and cost-efficient way for frequent in situ measurements, even repeated in time on the same target during the growth cycle. Since it has been proven for a long time that in several crops, the leaf N content is strongly correlated to the leaf chlorophyll (Chl) level [8,9], optical sensors able to detect Chl can be in principle good estimators of the plant N status. SPAD and N-Tester meters are among the most commonly used tools in many plant species [10,11,12,13,14] including cabbage [15]. However, some authors warned about the possibility that the relationship between chlorophyll meter readings and the leaf N content may be significantly affected by environmental factors and leaf characteristics [16].

The Dualex sensor, combining leaf transmittance and fluorescence measurements, was also introduced to control fertilization-related compounds in crops [17,18,19] and ornamental woody species [20,21,22]. It provides indices of both leaf flavonols and chlorophyll that are known to be compounds inversely and positively correlated to N availability, respectively [8,23]. In addition, the Dualex device provides the nitrogen balance index (NBI), which is the chlorophyll/flavonols ratio (related to nitrogen/carbon allocation), that can be used as a proxy of the nitrogen status of plants [24]. It is expected to be a more robust and sensitive index of the plant N status than those considering only a single class of compounds.

Compared to SPAD, the Dualex sensor presented a linear response to increasing leaf Chl content [25] and was more time efficient and stable in monitoring crop nitrogen status of rice [26]. The Flav index of flavonols had a better impact on monitoring and diagnosis of crop nitrogen nutrition as compared to the Chl index and NBI and was less dependent on the growth stage of rice [26]. Gabriel et al. [27] reported that leaf-clip chlorophyll sensors, as SPAD and Dualex, exhibited comparable results, however additional information such as flavonols can improve maize N status information. In potatoes, Ben Abdallah et al. [11] showed that flavonols-based indices, as provided by the Dualex, were more suitable than indices based on leaf transmittance and reflectance in assessing the crop nitrogen status. The highest capability of Dualex in estimating N content in rice leaves with respect to SPAD and a new digital imaging method was also observed [12].

The results obtained by Dong et al. [28] on four different plant species suggested that the Dualex is a better choice for collecting leaf chlorophyll measurements for different crops in the field, compared with the SPAD-502 and the CCM-200. Dong et al. [29] also focused on the open question about what is the optimal growth stage to define the plant N estimation model based on the Dualex sensor. In maize, they found that a modified NBI to take into account the period of growing after sowing strongly improved its relationship with the plant N status.

Optical sensors could also be useful to predict the yield of maize, improving the prediction accuracy as the crop development progressed [27], wheat [10], bell pepper [13], or cabbage [24,30].

The aim of this study was to evaluate the applicability of the Dualex optical sensor to determine the nitrogen status of cabbage plants and predict cabbage yields. This was performed on the Typhoon hydrid that is a late (120–130 days to maturity), high yielding white head cabbage cultivar used for both processing and long-term storage.

Optical monitoring was performed during two periods of the season, that is at the beginning of July (39 and 49 days after transplanting in 2018 and 2019, respectively) and of October (harvest) in order to check the effect of the plant growing stage on the N prediction.

## 2. Materials and Methods

### 2.1. The Dualex Optical Sensor

The Dualex Scientific+ (Force-A, Orsay, France) leaf-clip device was previously described by Goulas et al. [31]. It is an active battery driven portable sensor, measuring a sample spot of 6 mm diameter. The head contains five different LEDs, one emitting in the UV-A at 375 nm, two visible-emitting LEDs at 520 and 650 nm and two NIR-emitting LEDs at 710 and 850 nm. Signal detection occurs through a filtered PIN photodiode. The acquired data can be displayed on a LCD screen, stored in the internal flash memory and downloaded via a USB interface.

The sensor provides a Chl index determined as leaf transmittance in the red-edge of the chlorophyll absorption band at 710 nm with respect to a reference signal in the near-infrared at 850 nm that depends exclusively on leaf scattering [25].

Indices of epidermal flavonols or anthocyanins are determined according to the chlorophyll fluorescence (ChlF) excitation screening method [32,33]. Chlorophyll located in the leaf mesophyll, below the compounds of interest present in the leaf epidermis, emits bright fluorescence signals proportional to the excitation light received. This is dependent on the concentration of epidermal compounds that attenuate the excitation light reaching the chloroplasts at specific spectral bands. For this, ChlF is reduced proportionally to the presence of flavonols in the epidermis, absorbing in the UV-A spectral region, when excitation at 375 nm is used. Analogously, epidermal anthocyanins will reduced ChlF proportionally to their concentration when excitation at 520 nm is used. ChlF signals under excitation at 650 nm, at which the epidermal layer appears transparent (no attenuation) are used as reference.

The index of flavonols, Flav, provided by the Dualex sensor is calculated as the logarithm of the ratio between the ChlF excited at 650 nm and that at 375 nm:

Flav = log(ChlF_650_/ChlF_375_) (1)

while the nitrogen balance index (NBI) is given by

NBI = Chl/Flav(2)

as the ratio between the chlorophyll and flavonols indices.

### 2.2. Experimental Site and Data Acquisition

Field experiments were conducted in 2018 and 2019 at the Research Institute of Horticulture in Skierniewice, central Poland (51°57′49.64″ N, 20°10′23.77″ E, altitude 133 m). Experiments were conducted on a sandy-loam soil composed of 68% sand (0.1–1 mm grain size), 19% silt (0.1–0.2 mm), and 13% clay (<0.02 mm). The soil was characterized by pH 6.5, 1.16% content of organic matter and available water capacity of 12.5 mm H_2_O 0.1 m soil^−^^1^.

Cabbage (*Brassica oleracea* L. var. *capitata* subvar. *alba*) cv. Typhoon F1 was grown from transplants and planted in the field on 25th May 2018 and 3rd June 2019. The experiment involved a randomized complete block design (RCBD) with six replicates, each covering a 10 m^2^ area. Cabbage was planted at the distance of 56 × 60 cm (about 30 thousand ha^−^^1^). Nitrogen fertilization in the form of ammonium nitrate was applied one week before planting at the rates of 0, 100, and 200 kg N ha^−^^1^. A forth rate of 300 kg N ha^−^^1^ was supplied as the sum of two doses of 200 and 100 kg N ha^−^^1^, the first was applied along with the other rates as the pre-plant fertilization, whereas a second application was supplied as side dress fertilization about one month after transplanting to the field.

The phosphorus and potassium fertilization was applied each year according to the results of soil tests by bringing up the soil fertility to 80 mg P and 200 mg K per liter of soil. Drip irrigation was applied when soil moisture tension measured by a IRROMETER tensiometer (IRROMETER Co., Riverside, CA, USA) at the depth of 30 cm reached 0.04 MPa.

**Figure 1:**
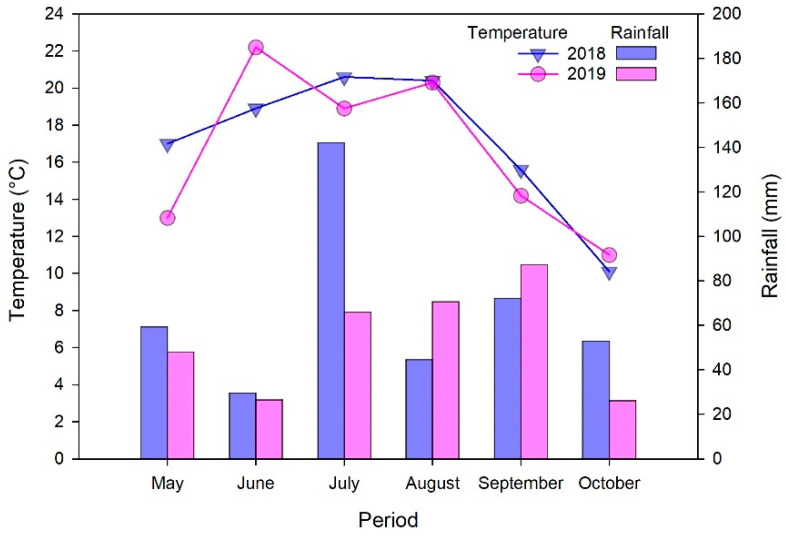
Monthly mean air temperature and monthly sum of precipitation recorded during the 2018 and 2019 seasons at the experiment site.

The monthly precipitation and average air temperature recorded during the two growing seasons are reported in Figure 1. The mean seasonal average air temperature was 18.4 °C and 17.6 °C in 2018 and 2019, respectively. The total precipitation during the growing season was 401 mm and 325 mm in 2018 and 2019, respectively.

In Situ Dualex leaf measurements were taken twice a year on 3 July 2018 and 22 July 2019, at 39 and 49 days after transplanting (DAT), respectively, and on 8 October 2018 and 7 October 2019, at 136 and 126 DAT, respectively. Measurements were taken on each plant (16 plants/plot) on the apical part of a fully developed leaf next to the head, recording data from both the adaxial and abaxial sides at about 2 cm from the leaf margin. For each leaf, the Chl and Flav were calculated as the average and the sum of the adaxial and abaxial values, respectively, then the NBI = Chl/Flav was determined.

For each treatment and replicate, one leaf/plant for 16 plants was measured and the average values of the sensor indices calculated. After measurements, the same leaves were used to determine the nitrogen content by using the Kjeldahl method (AOAC, 1990) [34]. Cabbage heads were harvested on 9th October 2018 and 8th October 2019. Healthy cabbage heads were separated into non-marketable and marketable according to European Union marketing regulations, CE 634/2006 [35], corresponding to cabbages with and without outer leaves, respectively.

For each replicate, the average leaf N was calculated over 8 samples and cabbage yields were the sum of weights of 16 samples.

### 2.3. Statistical Analysis

Statistical analysis and scatter plot curve fitting was performed with the SigmaPlot Program 14.0 (SigmaPlot for Windows, Systat Software, Inc., San Jose, CA, USA).

Leaf N content and cabbage yield data as function of the N rate applied in the RCBD model were statistically evaluated by means of Two Way ANOVA without replication, *p* values of <0.05 were considered statistically significant. Means under different N rates were compared by the all pairwise multiple comparison procedures (Holm–Sidak method).

The correlation among determined N content in cabbage leaves and Chl, Flav, and NBI indices, as well as between yields and NBI, were analyzed using linear regression and the coefficient of determination (R^2^).

## 3. Results

### 3.1. Fertilization Effect on Leaf N and Yield

The mean values of the cabbage leaf N content (%) determined by the Kjeldahl method for the different fertilization treatments at about one month and half after transplanting (July) and at harvest (October) and two seasons are reported in Table 1. Total and marketable yields for the two seasons under the four N rates are also shown (Table 1).

The statistical analysis showed that the difference in the mean values among the different levels of N was strongly significant at *p* < 0.001 for all cabbage yields and for most of the leaf N data, apart from the Oct 2019 values for which *p* = 0.005. In any case, variability of mean values among blocks was not significant with *p* between 0.05 and 0.995.

As expected, both leaf N content and yields increased with N rates from 0 to 300 kg N ha^−^^1^. Within each period, mean values among treatments were significantly (*p* < 0.05) different except for the leaf N at the two highest N doses of July 2018 and the leaf N of October 2019.

Leaf N reached values close to 4.7% and 2% in July and October, respectively. The ratio between the 2018 and 2019 values of both total and marketable cabbage yields was about 1.5 for the 300 kg N ha^−^^1^ and increased with decreasing the N rate to about 2.5 at 0 kg N ha^−^^1^.

### 3.2. Relationship between Leaf N Content, Yield and Optical Indices

**Figure 2:**
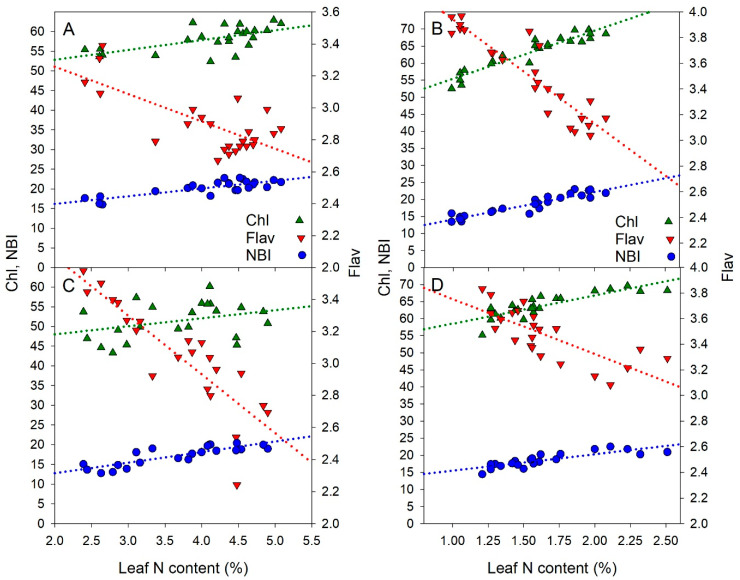
The relationship between chlorophyll (Chl), flavonols (Flav) and NBI optical indices and the nitrogen content in cabbage leaves in 2018: July (**A**) at 39 DAT, October (**B**) at 136 DAT and 2019: July (**C**) at 49 DAT, October (**D**) at 126 DAT.

Figure 2 shows the relationship between the chlorophyll (Chl), flavonols (Flav) and NBI optical indices and the N content in the cabbage leaves. Each couple of data was fitted by a linear regression model whose parameters are reported in Table 2. The lower determination coefficients for Chl occurred in July at head formation (R^2^ = 0.39, 2018 and 0.12, 2019) as compared to those in October before harvest (R^2^ = 0.89, 2018 and 0.70, 2019) (Table 2).

In the case of Flav, a high negative relationship between the flavonols index and the leaf N content was observed in both years of the study at both development stages of cabbage, and the determination coefficient ranged from 0.50 to 0.83 (Table 2). 

The highest correlations were found between NBI and the leaf N content with much less dispersion of points around the linear regression line with respect to the other indices (Figure 2). Depending on the year and growth stage, the determination coefficient ranged from 0.66 to 0.87 (Table 2).

**Figure 3:**
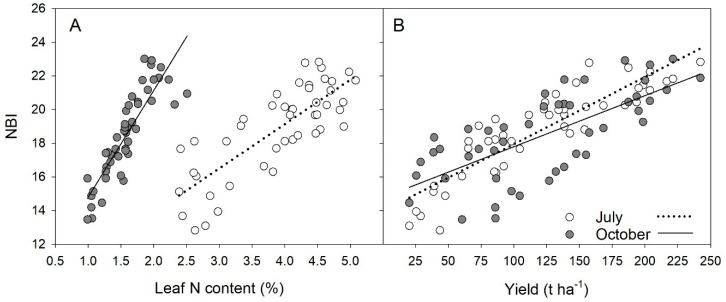
Relationship between NBI and cabbage leaf N content (**A**) and between NBI and total cabbage yield (**B**) for the two consecutive years of the experiment grouped according to the sampling date, in July (39 and 49 DAT, in 2018 and 2019, respectively) and October (136 and 126 DAT, in 2018 and 2019, respectively).

Figure 3A reports the relationship between NBI and the total cabbage leaf N content for the two consecutive years of the experiment grouped according to the sampling date, in July and October. The determination coefficient was 0.64 and 0.76 for July and October, respectively (Table 2). The regression model for NBI versus leaf N in July was superior to that for Chl versus leaf N, while in October the two models were similar (Table 2).

The average values of the Dualex indices as function of N rates for the two seasons and growing periods are reported in Supplementary Figure S1. It can be seen that generally NBI was able to discriminate the different N treatments better than Chl.

The NBI was also found to be correlated to cabbage yield, as shown in Figure 3B for the two-year merged data. The July and October data were both fitted by linear functions slightly differing in slope and intercept (Table 2). At harvest, points were much more scattered (R^2^ = 0.50) than in July. Results of the fitting analysis for the NBI versus single-year cabbage yield at two growing stages are reported in Table 3.

## 4. Discussion

The above results showed a significant effect of nitrogen fertilization on both total and marketable yields of cabbage, with maximal values reached at 300 kg N ha^−^^1^, which is a common N dose for cabbage, in both years (Table 1). This confirms the results of earlier studies, which found a positive effect of nitrogen fertilization on yields of the same Typhoon cabbage hybrid and the similar Transam hybrid [24,36].

These cabbage hybrids can produce significant high yields but largely dependent on the season. For each N rate, both total and marketable cabbage yields were much lower in 2019 compared to 2018. They reduced of about 38% at 0 kg N ha^−^^1^ and up to 67% at 300 kg N ha^−^^1^. This was likely due to adverse weather conditions of drought and high temperature during the 2019 growing season (Figure 1). Clearly, under dry conditions and low air humidity, irrigation was not enough to ensure high yields of cabbage in 2019.

Siedel et al. [37] also reported higher yields of the Typhoon cabbage variety under more wet and chilly conditions at the beginning of the growing period with respect to drier and warmer seasons. Cabbage can be grown under wide different environmental conditions, but prefers cool and wet climate with mean daily temperature of about 17 °C, minimal and maximal temperature of 10 °C and 24 °C, respectively, and 60–90% of mean relative humidity.

Total nitrogen contents in the leaves of cabbage were positively correlated to the N rates. They reached the highest value of about 4.8% at 300 kg N ha^−^^1^ around 39 and 49 DAT, depending on the season. For both years and all N applications, the content of nitrogen in cabbage leaf in July was more than twice that in October, when most of the N available in the soil was reduced by the large plant consumption during the head developing phase (Table 1). In fact, the highest leaf N content appeared at the early stage of plant growing when the highest N uptake occurs, then leaf N decreased towards harvest due to dilution [38]. Since the level of N accumulated by the cabbage plants during the two seasons was similar, the lower yield in 2019 indicates a reduced N use efficiency during this year due to more stressful conditions.

### 4.1. Correlation between Dualex Indices and Leaf N

Leaf nitrogen content under different N fertilizer rates was positively correlated to Chl and NBI indices, while it was negatively correlated to the Flav index (Figure 2). Results of both years of the experiment showed that these correlations were dependent on the growth stage of the cabbage plants (Table 2).

The Chl vs. leaf N relationship in July was more flat than that in October. The slope of the regression in October was more than 4–5 time that in July. Although in July there was an excess of leaf N, the chlorophyll content did not increase much. Chlorophyll concentration did not follow the same pattern as leaf N. While in October, with lower availability of N, the correlation between Chl and leaf N was improved.

The inverse correlation found between Flav and the leaf N content is consistent with those obtained with rice by Zhang et al. [26], with cabbage by Agati et al. [24], in bell pepper [13] and also in cucumber [6], turfgrasses [39] or ornamental plants [20,22]. 

Gabriel et al. [27] reported that differences in the Flav content between fertilizer treatments were larger in the ear leaf of maize and increased with time, making it easier to detect fertilizer deficiencies measuring this leaf. 

Since NBI combines the opposite behavior of Chl and Flav with N, it maximizes the N treatment differences. This was also evident by plotting the average values of indices against the N rates (Supplementary Figure S1). Other authors [40,41] reported that NBI represents a more robust proxy of the plant N status than Chl or Flav alone and that the NBI power in discriminating different N treatments increased with time [27]. Accordingly, we also observed a larger span of NBI in October with respect to July.

Considering the two years of investigation together, it resulted that in July the relationship between NBI and total leaf N content was more flat and dispersed than in October (Figure 3A). The two linear regressions had a similar intercept but the slope in October was more than double that in July. This varying behavior is likely due to the different allocation of carbon to chlorophyll synthesis during the two growing stages considered. Similarly, Zhang et al. [26] reported that in rice there was a good correlation between NBI and different nitrogen nutrition indices and that the determination coefficient varied according to the growing period. 

In cucumber, Padilla et al. [40] showed that the relationship between NBI and the nitrogen nutrition index (NNI) evaluated over two seasons was better during the reproductive period than at harvest or during the vegetative period. It seems, therefore, that for different species there is an optimal timespan to apply the Dualex indices for the plant N status determination.

Because of the large variability observed in the NBI versus leaf N relationship as function of the cabbage growing stage, the optical monitoring of cabbage leaves should be repeated at different times during the season. This is fundamental for merging the non-destructive approach into a decision making procedure for fertilization. Yet, seasonal variation of data makes a general calibration curve of the Dualex sensor not sufficiently precise in establishing a deficient or excessive crop N status.

A relative approach using reference well-fertilized plot without N limitations is then suggested and should be tested on cabbage crops in the future. This would reduce most of the possible interference on the sensor readings due to factors other than N fertilization [9]. 

Alternatively, absolute sufficiency values of the sensor readings can be determined on the basis of yield or the NNI [42]. The adequate optical indices for maximum yield are defined by a segmented linear regression analysis of the yield versus index scatter plot, in which an initial linear increase is followed by a plateau. The breaking point of this relationship defines the sufficiency value.

From Figure 3B, it can be envisaged that within the N rate range applied no saturation of the yield versus NBI occurred. Therefore, in our experiment we could not calculate the sufficiency index for maximum cabbage yield.

### 4.2. Correlation between NBI and Yield

In our study, we also found a correlation between NBI and yield of cabbage (Figure 3B), with both positive regressions for the early detection (July) and at harvest (October). The large scattering of points observed at harvest was clearly due to a seasonal effect, since the relationship evaluated for single-year was satisfactory (R^2^ of 0.88 and 0.80, in 2018 and 2019, respectively) (Table 3).

The early measurements of the NBI index in July can be useful for predicting yield of cabbage in advance and then allowing enough time to change the crop management if needed. In agricultural practice, split nitrogen doses are used to avoid nitrogen losses through leaching. The first dose is used as pre-plant fertilization, while the second is applied at about a month after transplanting, which in the case of cabbage is at the beginning or mid-July. Knowing the nutritional status of plants makes it easier to take a decision on the application time and the amount of the next N dose. This can also be reduced when unfavorable weather conditions preclude reaching the maximum yield, then favoring N leaching.

To notice that the correlation of NBI versus yield measured at 39 and 49 DAT in 2018 and 2019, respectively, fitted with linear models (Table 3), was superior to that between the NDVI, the normalized difference vegetation index, and yield at the early stages of cabbage growth [30]. Close to harvest, our methodology and that of Ji et al. [30] gave similar results concerning the quality of the optical indices versus yield relationship.

Here, we did not verify if, and how much, the N fractioning based on NBI detection early in the growing season could manage, at an intermediate stage, the fertilization to increase the cabbage yield. This could be matter of future work.

## 5. Conclusions

Our study proved that the Nitrogen Balance Index provided by the Dualex sensor, equal to the ratio of the simultaneous estimate of leaf chlorophyll and flavonols, can be a valuable innovative tool for the sustainable assessment of the N status in Brassica oleracea.

The in-field optical sensing performed early in the season, in between 39 and 49 days after transplanting, could be practically useful in order to manage supplemental N fertilization adjustments.

The use of NBI to forecast cabbage yield can have significant advantages for the economic trading and food production monitoring and can contribute to reducing the environmental contamination due to nitrate leaching.

In the application of the method, it must be considered that flavonols accumulation in the leaves is critically dependent on the solar irradiation received [32]. For this, in the assessment of the crop N status care must be paid to optically sampling leaves with similar sun exposure.

Mapping of a whole field crop by using the Dualex sensor is possible [24] and can represent an original improvement of the agronomic practices for the *Brassica oleracea* cultivation in order to deliver nitrogen at the right place, at the right time and in the right amount to increase the fertilizer use efficiency. 

One limit of the Dualex sensor is related to the small leaf area of sampling. This restriction can be overcome by increasing the number of measurements per sample or by using other devices based on the same fluorescence acquisition method, as the Multiplex (Force-A, Orsay, France), that integrate the detected signals from the leaf lamina on a diameter spot of up to 8 cm. Furthermore, since the Multiplex sensor operates with a reflection geometry, it can be also applied for measurements on cabbage heads for which the use of the Dualex is not possible because of their tightly overlaid leaves. In this way, the technique, besides the evaluation of the crop N status, can provide information on the cabbage head content of bioactive compounds (flavonols) and chlorophyll important for the green appearance and storability of leafy vegetables and to better characterize their nutritional value.

## Figures and Tables

**Table 1:** Mean values ±SD (*n* = 6) of cabbage leaf N content determined by the Kjeldahl method (J-July, O-October) and total and marketable cabbage yields as function of N rate ^1^.

N Rate (kg ha^−1^)	Leaf N Content (%)				Total Yield (t ha^−1^)		Marketable Yield (t ha^−1^)	
	2018		2019		2018	2019	2018	2019
	J	O	J	O				
0	2.96 ± 0.66c	1.04 ± 0.04d	2.86 ± 0.40d	1.34 ± 0.11b	87 ± 14d	33 ± 8d	46 ± 12d	18 ± 5d
100	4.14 ± 0.29b	1.44 ± 0.16c	3.33 ± 0.60c	1.51 ± 0.16b	145 ± 15c	65 ± 18c	85 ± 6c	42 ± 16c
200	4.54 ± 0.23ab	1.70 ± 0.13b	3.97 ± 0.18b	1.79 ± 0.46ab	191 ± 17b	105 ± 20b	116 ± 10b	67 ± 18b
300	4.76 ± 0.22a	1.95 ± 0.09a	4.77 ± 0.35a	2.13 ± 0.31a	209 ± 16a	141 ± 10a	137 ± 16a	92 ± 9a

^1^ Within each column, values marked by the same lowercase letter do not differ significantly at *p* = 0.05 according the Holm-Sidak test.

**Table 2:** Equations and R^2^ of the regressions between Chl, Flav, NBI indices and the leaf N content and between NBI and total cabbage yield.

Period	Chl vs. Leaf N	Flav vs. Leaf N	NBI vs. Leaf N	NBI vs. Yield
July 2018	y = 2.49x + 47.9 R^2^ = 0.394 *	y = −0.171x + 3.6 R^2^ = 0.523 *	y = 1.96x + 12.3 R^2^ = 0.663 **	
July 2019	y = 2.06x + 43.9 R^2^ = 0.119	y = −0.367x + 4.4 R^2^ = 0.751 **	y = 2.66x + 7.47 R^2^ = 0.712 **	
July 2018 + 2019	y = 3.24x + 42.3 R^2^ = 0.258 *	y = −0.27x + 4.02 R^2^ = 0.633 **	y = 2.64x + 8.58 R^2^ = 0.64 *	y = 0.04x + 13.95 R^2^ = 0.801 **
October 2018	y = 14.10x + 41.3 R^2^ = 0.889 **	y = −0.81x + 4.75 R^2^ = 0.835 **	y = 8.06x + 6.08 R^2^ = 0.873 **	
October 2019	y = 8.3x + 50.2 R^2^ = 0.705 **	y = −0.435x + 4.18 R^2^ = 0.567 *	y = 4.79x + 10.72 R^2^ = 0.696 *	
October 2018 + 2019	y = 11.1x + 45.8 R^2^ = 0.778 **	y = −0.62x + 4.47 R^2^ = 0.693 **	y = 6.34x + 8.45 R^2^ = 0.76 **	y = 0.03x + 14.76 R^2^ = 0.497 **

* significant difference at *p* < 0.05, ** *p* < 0.01.

**Table 3:** Coefficient of determination (R^2^) for the linear correlation between NBI and single-year cabbage yield at two growing stages.

Year	Days after Transplanting	R^2^
2018	39	0.71
	136 (harvest)	0.88
2019	49	0.76
	126 (harvest)	0.80

## Data Availability

The data presented in this study are available on request from the corresponding author.

## Supplementary Materials

The following are available online at https://www.mdpi.com/1424-8220/21/2/482/s1, Figure S1: Average values (±SD) of the NBI (grey bars), Chl (white bars) and Flav (striped bars) Dualex indices as function of the N rates (*n* = 90) indices in 2018: July (A) at 39 DAT, October (B) at 136 DAT and 2019: July (C) at 49 DAT, October (D) at 126 DAT. For each index, means followed by the same letter do not differ significantly at *p* = 0.05 according the Holm-SidakTukey test.
