# Supplementary material for: Application of a Combined Transmittance/Fluorescence Leaf Clip Sensor for the Nondestructive Determination of Nitrogen Status in White Cabbage Plants
Source: Sensors (Basel). 2021 Jan 12;21(2):482. doi: 10.3390/s21020482 (PMC7827347; doi:10.3390/s21020482)
Supplement: Supplementary file 1 [file sensors-21-00482-s001.pdf]

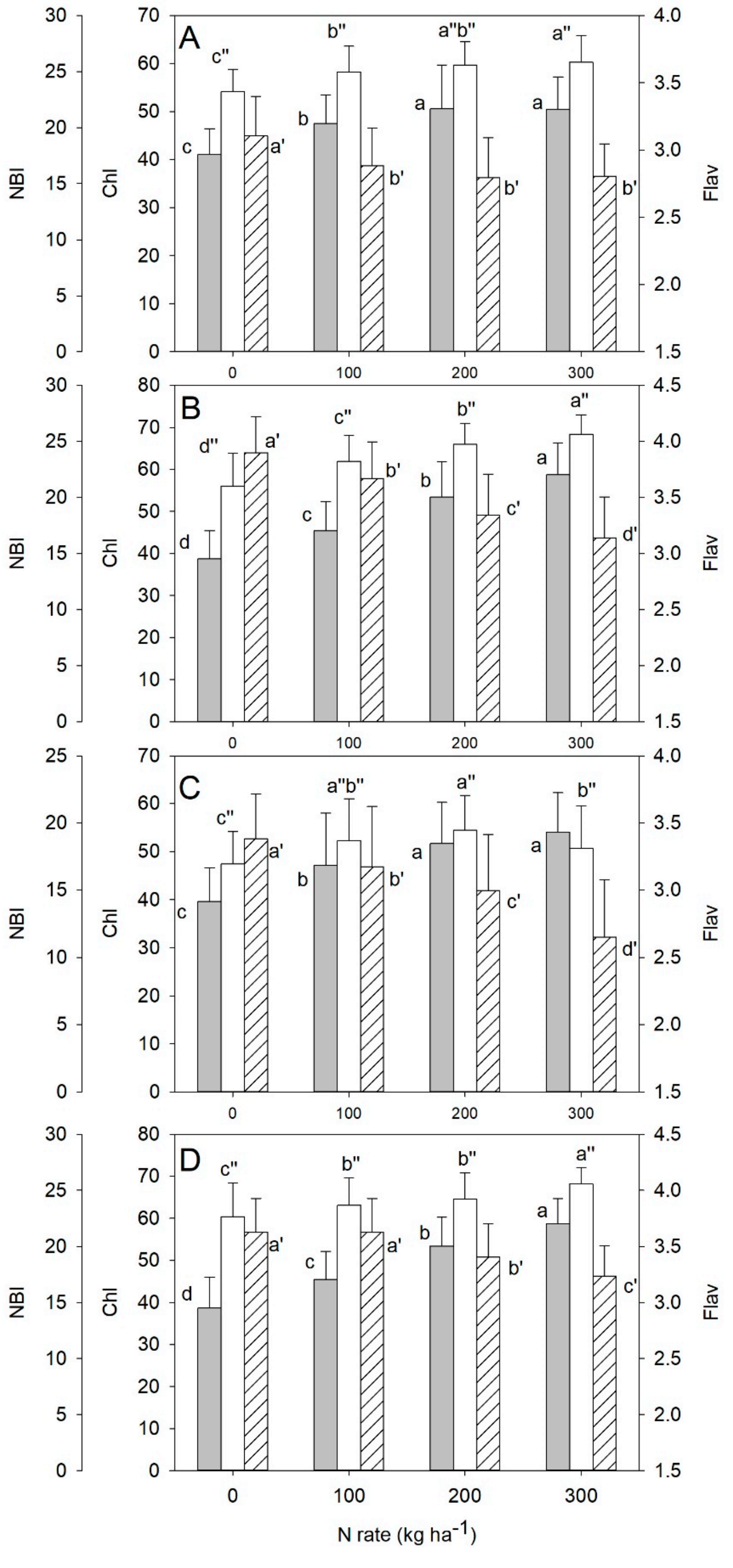

Figure S1. Average values (±SD) of the NBI (grey bars), Chl (white bars) and Flav (striped bars) Dualex indices as function of the N rates (n=90) indices in 2018: July (A) at 39 DAT, October (B) at 136 DAT and 2019: July (C) at 49 DAT, October (D) at 126 DAT. For each index, means followed by the same letter do not differ significantly at p=0.05 according the Holm-SidakTukey test.
